# Evaluation of intraretinal migration of retinal pigment epithelial cells in age-related macular degeneration using polarimetric imaging

**DOI:** 10.1038/s41598-017-03529-8

**Published:** 2017-06-09

**Authors:** Masahiro Miura, Shuichi Makita, Satoshi Sugiyama, Young-Joo Hong, Yoshiaki Yasuno, Ann E. Elsner, Shigeo Tamiya, Rintaro Tsukahara, Takuya Iwasaki, Hiroshi Goto

**Affiliations:** 1 Department of Ophthalmology, Tokyo Medical University, Ibaraki Medical Center, Ami, Japan; 2 Department of Ophthalmology, Tokyo Medical University, Tokyo, Japan; 3 Computational Optics Group, University of Tsukuba, Tsukuba, Japan; 4 Tomey Corporation, Nagoya, Aichi Japan; 5 School of Optometry, Indiana University, Bloomington, IN USA; 6 Department of Ophthalmology and Visual Sciences, University of Louisville, Louisville, KY USA

## Abstract

The purpose of the present study was to evaluate the intraretinal migration of the retinal pigment epithelium (RPE) cells in age-related macular degeneration (AMD) using polarimetry. We evaluated 155 eyes at various AMD stages. Depolarized light images were computed using a polarization-sensitive scanning laser ophthalmoscope (PS-SLO), and the degree of polarization uniformity was calculated using polarization-sensitive optical coherence tomography (OCT). Each polarimetry image was compared with the corresponding autofluorescence (AF) images at 488 nm (SW-AF) and at 787 nm (NIR-AF). Intraretinal RPE migration was defined by the presence of depolarization at intraretinal hyperreflective foci on PS-SLO and PS-OCT images, and by the presence of hyper-AF on both NIR-AF and SW-AF images. RPE migration was detected in 52 of 155 eyes (33.5%) and was observed in drusenoid pigment epithelial detachment (PED) and serous PED with significantly higher frequencies than in other groups (P = 0.015). The volume of the migrated RPE cluster in serous PED was significantly correlated with the volume of the PED (R^2^ = 0.26; P = 0.011). Overall, our results showed that intraretinal RPE migrations occurred in various AMD stages, and that they occurred more commonly in eyes with serous and drusenoid PED.

## Introduction

In most developed countries, age-related macular degeneration (AMD) remains a leading cause of severe visual loss in older patients^1^. Alterations of the retinal pigment epithelium (RPE) can serve as important indicators of AMD development^2–5^. For instance, the RPE may exhibit various cellular stress responses, including sloughing, proliferation, entombing, shedding, and migration^2, 3^. Among these stress responses, intraretinal RPE migration into the neurosensory retina has been reported as an important precursor of chorioretinal atrophy^6–9^ and of the RPE cell death pathways^2^. Clinical studies using standard optical coherence tomography (OCT) have suggested that intraretinal hyperreflective foci (HRF) are good candidates for intraretinal RPE migration^6, 9–12^. HRF have been reported in various retinal diseases, including AMD^6–8, 10–15^, retinitis pigmentosa^16^, diabetic retinopathy^17, 18^, and retinal vein occlusion^19, 20^. Previous studies have proposed several potential origins of the HRF, including, lipoprotein aggregation^18–20^, inflammatory cells^17^, and RPE migration^6, 10–12^. In AMD eyes, HRF with drusen^6, 10^ is reported to be associated with the development of geographic atrophy^6–8^. Furthermore, the number of intraretinal HRF observed in AMD was shown to decrease after treatment^13^, and the presence of intraretinal HRF before treatment was correlated with poor visual outcomes^14^. Histopathological analyses have confirmed the presence of RPE migration in the intraretinal HRF of AMD patients^21, 22^. Additionally, the spatial association between intraretinal HRF and hyperpigmentation using color fundus photography was consistent with the hypothesis of intraretinal RPE migration as an origin of HRF in AMD^23^. Despite these reports, the origin of HRF is still unclear, in part because OCT has an insufficient spatial resolution and a limited capability to investigate HRF constituents and intraretinal RPE migration *in vivo*.

Polarimetry techniques have been developed to selectively characterize the different layers of the retina. In the human retina, three distinct physical properties, involving depolarization, birefringence, and polarization preservation, can be distinguished using polarimetry imaging^24^. The multiple light-scattering properties of melanin in tissue induce depolarization^25^, and depolarization has been observed in melanin-containing structures, such as the RPE, melanocytes, or melanin-containing inflammatory cells^25–32^. Imaging with a polarization-sensitive scanning laser ophthalmoscope (PS-SLO) provides an *en face* distribution of the polarization properties of the retina^26, 33, 34^. Polarization-sensitive optical coherence tomography (PS-OCT) is a functional extension of OCT technology that allows the acquisition of three-dimensional retinal information including polarization properties^24^. In PS-OCT, the depolarization or the polarization scramble of the tissue can be evaluated as a function of the degree of polarization uniformity (DOPU)^35^. DOPU numerical values range from 0.0–1.0, and lower values indicate the presence of a depolarization or a polarization scramble^35^. PS-OCT has enabled the detection of depolarization in HRF^31^. Furthermore, PS-OCT has been used to assist with the evaluation of RPE changes in AMD^25–32^. However, PS-OCT has important limitations for the detection of intraretinal RPE migration. HRF depolarization can be induced at several locations such as at the RPE, melanocytes, melanin-containing inflammatory cells, and hard exudates^24–32, 36^. Therefore, the presence of depolarization at HRF locations does not necessarily indicate the presence of intraretinal RPE migration.

To overcome this limitation, we combined polarimetry imaging with autofluorescence (AF) imaging. Clinical AF imaging techniques have been previously used to monitor RPE changes. Hyper-AF in short wavelength AF imaging (SW-AF; excitation 488 nm, emission > 500 nm) is thought to originate from sheared photoreceptor outer segments^37^, from lipofuscin in the RPE, or from melanolipofuscin, which is a substance composed of melanin and lipofuscin in the RPE^38, 39^. Hyper-AF in near infrared wavelength AF imaging (NIR-AF; excitation 788 nm, emission > 800 nm) is thought to originate from melanin or melanolipofuscin in the RPE, from melanin in melanocytes, or from melanin-containing inflammatory cells^40, 41^. Focal peaks of hyper-AF in NIR-AF or SW-AF images have been attributed to vertically superimposed fluorescent cells resulting from intraretinal RPE migration^42^. The concomitant confirmation of HRF depolarization with hyper SW-AF and hyper NIR-AF images at corresponding locations might confirm the presence of RPE migration by demonstrating the simultaneous existence of melanin and lipofuscin, or of melanin and melanolipofuscin. In the present study, we investigated the presence of intraretinal RPE migration in AMD using PS-OCT, PS-SLO, NIR-AF, and SW-AF. Furthermore, we correlated these imaging findings with the presence of pathophysiological markers of AMD.

## Results

**Figure 1:**
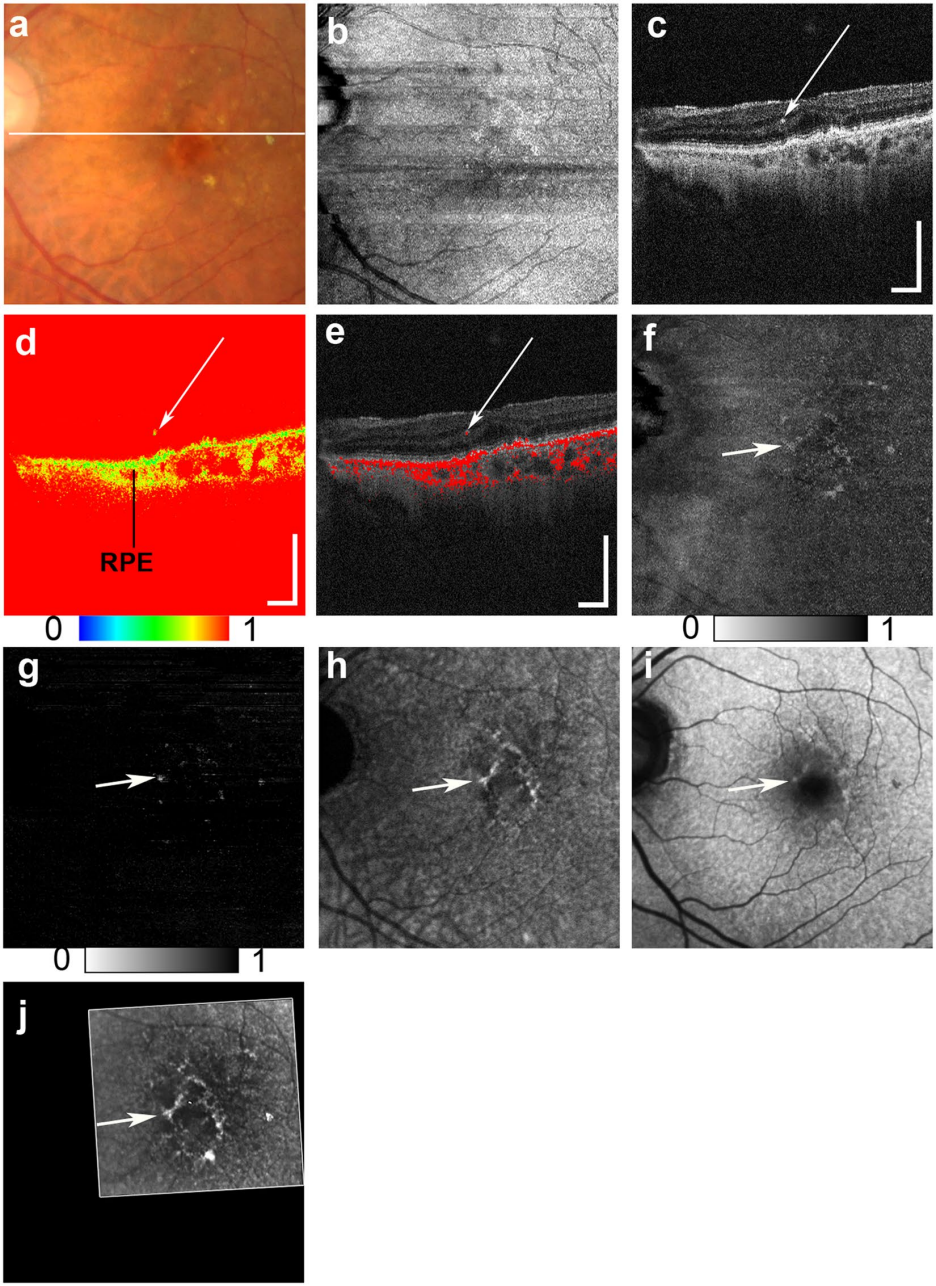
Multimodal imaging of early to intermediate age-related macular degeneration (AMD) from the left eye of an 81-year-old female. The white line in the color fundus photograph (**a**) indicates the scan line of the polarization sensitive optical coherence tomography (PS-OCT) B-scan images (**c,d,e**). An *en face* projection of the standard OCT image (**b**). A standard OCT B-scan image (**c**) showing hyperreflective foci (HRF; white arrow). A pseudocolor image of the corresponding degree of polarization uniformity (DOPU) B-scan image (**d**) and composite DOPU B-scan OCT images **(e)** showing depolarization at the HRF (white arrows). The scale bars represent 500 μm × 500 μm. An *en face* projection image of the minimum DOPU along the whole depth (**f**) and an *en face* projection of the minimum DOPU anterior to the retinal pigment epithelium (**g**), which showed depolarization at corresponding locations of HRF (white arrows). The autofluorescence (AF) images at 787 nm (NIR-AF) (**h**) and 488 nm (SW-AF) (**i**) show hyper-AF at corresponding locations of HRF (white arrows). A depolarized light image using the polarization sensitive scanning laser ophthalmoscope (PS-SLO) (**j**) shows depolarization at corresponding locations of HRF (white arrow).

**Figure 2:**
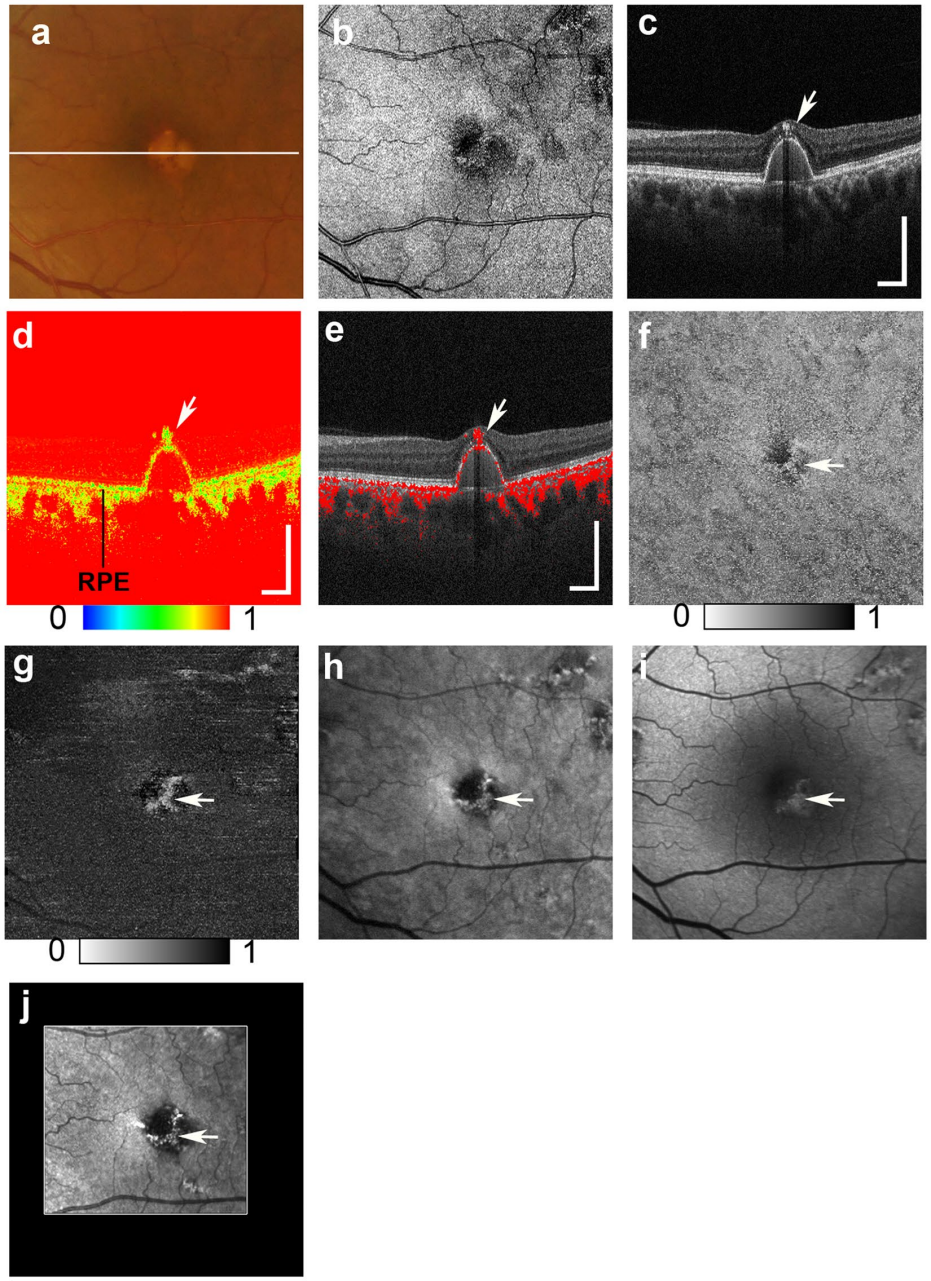
Multimodal imaging of drusenoid pigment epithelial detachment (PED) from the left eye of a 65-year-old male. The white line in the color fundus photograph (**a**) indicates the scan line of the PS-OCT B-scan images (**c,d,e**). An *en face* projection of the standard OCT image (**b**). The standard OCT B-scan image (**c**) showing HRF (white arrow). A pseudocolor image of the corresponding DOPU B-scan image (**d**) and composite DOPU B-scan OCT images **(e)** showing depolarization at the HRF (white arrows). The scale bars represent 500 μm × 500 μm. An *en face* projection image of the minimum DOPU along the whole depth (**f**) and an *en face* projection of the minimum DOPU anterior to the retinal pigment epithelium (**g**), which showed depolarization at corresponding locations of HRF (white arrows). The corresponding NIR-AF image (**h**) and SW-AF image (**i**) show hyper-AF at corresponding locations of HRF (white arrows). A depolarized light image using the PS-SLO (**j**) shows depolarization at corresponding locations of HRF (white arrow). All abbreviations used are as defined in the figure legend for Fig. 1.

**Figure 3:**
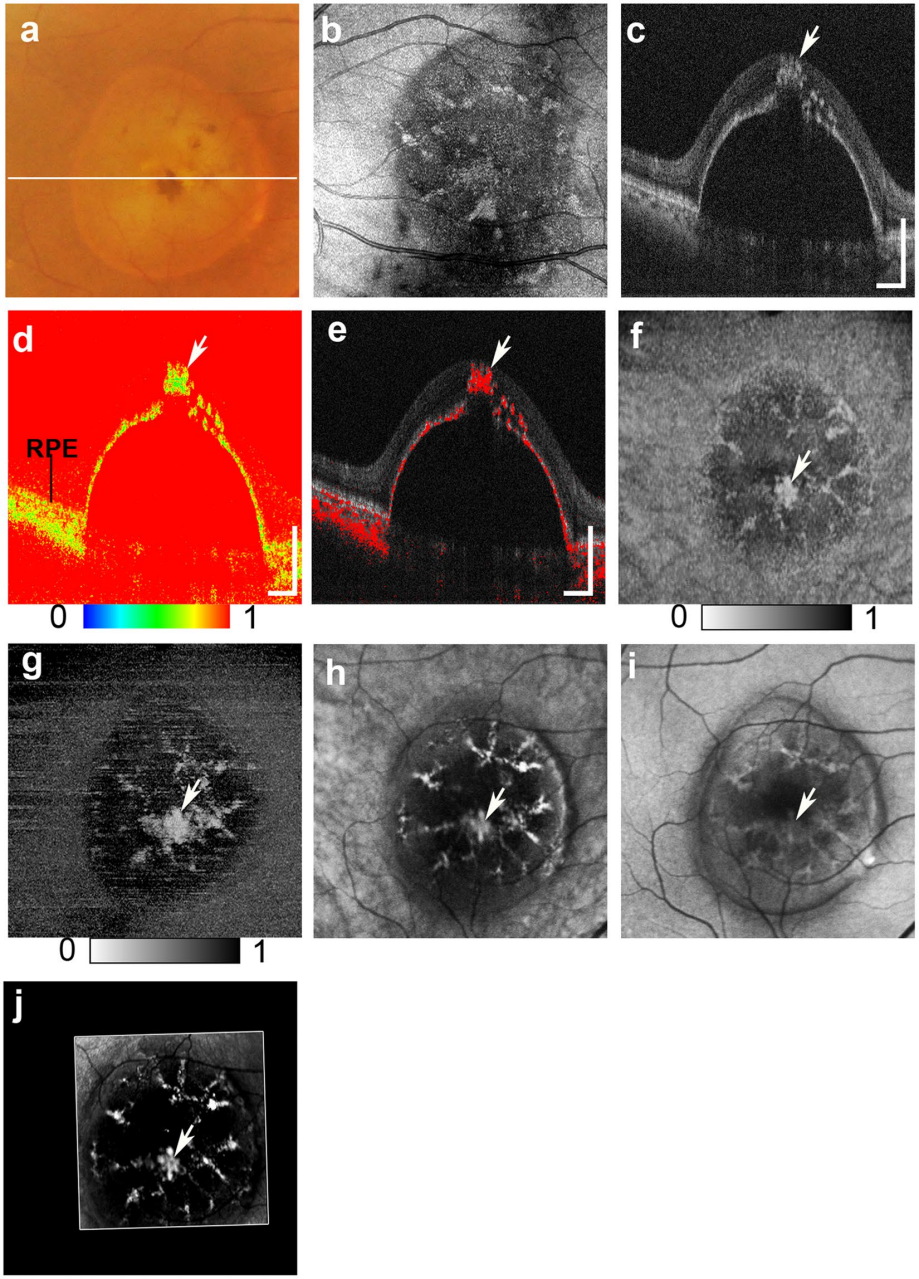
Multimodal imaging of serous PED from the right eye of a 70-year-old male. The white line in the color fundus photograph (**a**) indicates the scan line of the PS-OCT B-scan images (**c,d,e**). An *en face* projection of the standard OCT image (**b**). The standard OCT B-scan image (**c**) showing HRF (white arrow). A pseudocolor image of the DOPU B-scan image (**d**) and composite DOPU B-scan OCT images **(e)** showing depolarization at the HRF (white arrows). The scale bars represent 500 μm × 500 μm. An *en face* projection image of the minimum DOPU along the whole depth (**f**) and an *en face* projection of the minimum DOPU anterior to the RPE (**g**), which showed depolarization at corresponding locations of HRF (white arrows). NIR-AF image (**h**) and SW-AF image (**i**) show hyper-AF at corresponding locations of HRF (white arrows). A depolarized light image using the PS-SLO (**j**) shows depolarization at corresponding locations of HRF (white arrow). All abbreviation used are as defined in the figure legends for Figs 1 and 2.

**Figure 4:**
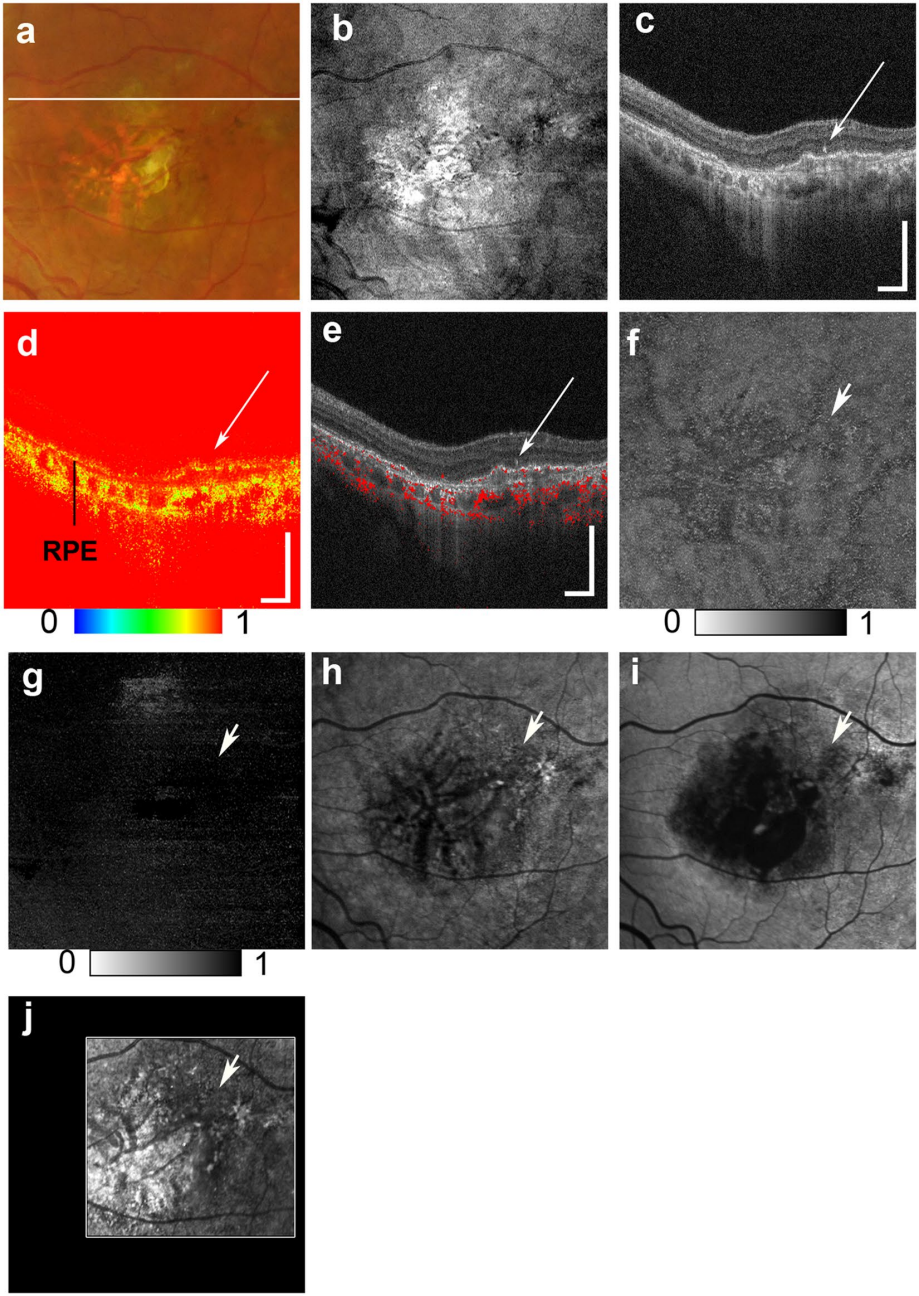
Multimodal imaging of end-stage AMD with subretinal fibrosis from the left eye of a 61-year-old male. The color fundus photography image (**a**) shows subretinal fibrosis on the macula, and the white line indicates the scan line of the PS-OCT B-scan images (**c,d,e**). The *en face* projection of a standard OCT image (**b**). The standard OCT B-scan image (**c**) shows HRF (white arrow). The DOPU B-scan image (**d**). Composite DOPU B-scan OCT images **(e)**. The scale bars represent 500 μm × 500 μm. An *en face* projection image of the minimum DOPU along the whole depth (**f**) and an *en face* projection of the minimum DOPU anterior to the RPE (**g**). The PS-OCT shows the absence of depolarization at HRF **(d,e,f,g**; white arrows). The absence of hyper-AF in NIR-AF (**h**), SW-AF (**i**) or depolarization in PS-SLO (**j**) at corresponding locations of HRF (white arrows) is consistent with the absence of RPE migration at HRF. All abbreviations used are as defined in the figure legends for Figs 1–3.

HRF with RPE migration were detected in 52 of the 155 eyes examined (33.5%), as evidenced by the concomitant presence of depolarization, hyper NIR-AF, and hyper SW-AF (Figs 1–3). The *en face* color fundus images typically seen in clinical practice showed the lateral location of the lesions (Figs 1a, 2a, 3a and 4a), but did not provide any three-dimensional information regarding the HRF locations in the retina, and more specifically, any information regarding the presence of RPE migration into the retinal layers. Similarly, the computed *en face* images obtained with OCT showed the location of the lesions and the main retinal blood vessels, but did not allow for the detection of RPE migration (Figs 1b, 2b, 3b and 4b). The standard OCT B-scan images were retinal cross sections that documented the presence and location of HRF within the layers of the retina (Figs 1c, 2c, 3c, and 4c), but did not provide information regarding the source of the hyperreflectivity. In contrast, when the PS-OCT cross-sectional DOPU images were plotted as pseudocolor, there were focal color changes that emphasized depolarization consistent with RPE changes, and the HRF could be localized within specific retinal layers in some eyes (Figs 1d, 2d, and 3d), but not in others (Fig. 4d). Location of HRF depolarization was readily determined with the composite DOPU B-scan OCT images (Figs 1e, 2e and 3e). *En face* projection images of the minimum DOPU anterior to the RPE clearly showed the distribution of HRF with RPE migration (Figs 1g, 2g and 3g). In many cases, similarities were found among the *en face* projection images of the minimum DOPU along the whole depth (Figs 1f, 2f and 3f), the depolarized light images (Figs 1j, 2j and 3j), and the NIR-AF images (Figs 1h, 2h and 3h).

Intraretinal RPE migrations were detected with different rates in each group (Table 1). The frequency with which intraretinal RPE migration was detected in the drusenoid pigment epithelial detachment (PED) group and in the serous PED group was significantly higher than in other groups (P = 0.015 and P = 0.0001, respectively; chi-square test; Table 1). HRF without RPE migration were observed (Fig. 4) with significantly higher rates in the remission and in the end-stage of exudative AMD groups compared with other groups (P = 0.031 and P = 0.0004, respectively; chi-square test; Table 1). The kappa value of interobserver agreement (R.T. and I.T.) for classification of HRF was 0.89.

**Table 1:** The number of eyes in each group that had HRF with an RPE migration and without an RPE migration.

Group	Total	HRF with RPE migration		HRF without RPE migration	
		Eyes	%	Eyes	%
Early- intermediate	14	5	35.7	0	0.0
Drusenoid PED	13	11	84.6	0	0.0
Serous PED	24	24	100.0	0	0.0
Remission	22	4	18.2	7	31.8
Fibrosis	69	4	5.8	35	50.7
GA	13	4	30.8	0	0.0

HRF, hyperreflective foci; RPE, retinal pigment epithelium; PED, pigment epithelial detachment, GA, geographic atrophy.

**Figure 5:**
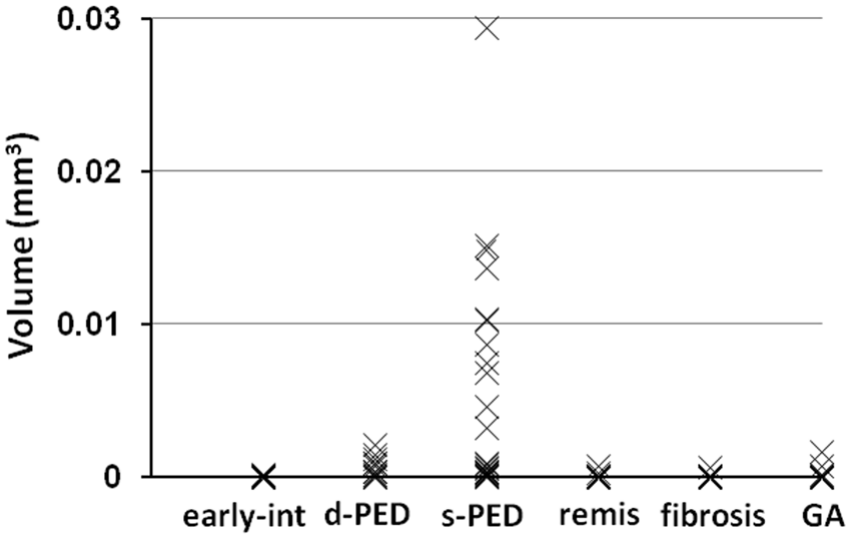
The distribution of the volumes of HRF with RPE in each AMD group. Early-int, early to intermediate AMD; d-PED, drusenoid PED; s-PED, serous PED; remis, remission stage of exudative AMD; fibrosis, the end-stage of AMD with subretinal fibrosis; GA, dry AMD with geographic atrophy. All abbreviations used are as defined in the figure legends for Figs 1–4.

**Figure 6:**
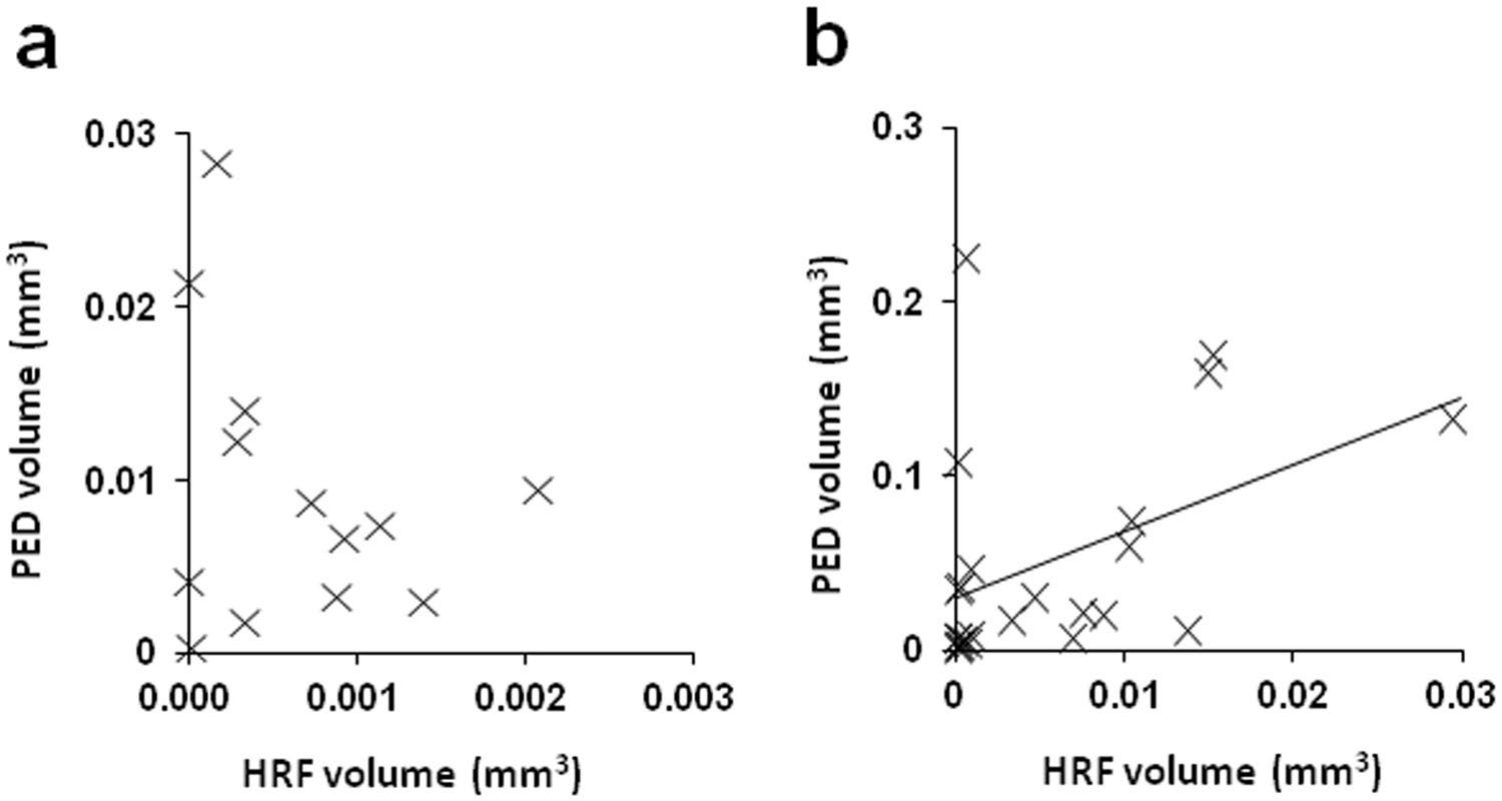
Scatterplots of the HRF and PED volumes. (**a**) Drusenoid PED group. (**b**) Serous PED group. All abbreviations used are as defined in the figure legends for Figs 1 and 2.

The mean HRF volumes in the drusenoid PED and serous PED groups were significantly larger than in other groups (P = 0.004; Kruskal–Wallis test, Mann-Whitney U test; Fig. 5, Table 2). Although the mean HRF volume in the serous PED group was larger than in the drusenoid PED group, the difference was not statistically significant (P = 0.12; Mann-Whitney U test; Fig. 5, Table 2). The mean PED volumes [mean ± standard deviation (range)] were 0.0094 ± 0.0081 mm^3^ (0.0004–0.028) and 0.0499 ± 0.0630 mm^3^ (0.0016–0.2249) in the drusenoid PED and serous PED groups, respectively. In the drusenoid PED group, the HRF volume did not show a significant correlation with the PED volume (R^2^ = 0.028; P = 0.20; Pearson’s correlation; Fig. 6). In the serous PED group, however, the HRF volume did show a significant positive correlation with the PED volume (R^2^ = 0.19; P = 0.034; Pearson’s correlation; Fig. 6).

**Table 2:** The volume of HRF with RPE in each group.

group	eyes	mean (mm^3^)	standard deviation	range (mm^3^)
Early- intermediate	14	0.000048	0.000099	0.000000–0.000346
Drusenoid PED	13	0.000638	0.000630	0.000000–0.002076
Serous PED	24	0.005340	0.007322	0.000004–0.029365
Remission	22	0.000043	0.000151	0.000000–0.000681
Fibrosis	69	0.000009	0.000071	0.000000–0.000592
GA	13	0.000197	0.000471	0.000000–0.001622

HRF, hyperreflective foci; RPE, retinal pigment epithelium; PED, pigment epithelial detachment; GA, geographic atrophy.

## Discussion

Intraretinal RPE migration has been reported to be a precursor of chorioretinal atrophy^6, 8^, and is an important finding in the RPE cell death pathways^2^. In the present study, intraretinal RPE migration was defined as the presence in HRF of melanin and lipofuscin, or melanin and melanolipofuscin. We characterized intraretinal RPE migration using a multimodal imaging protocol that included SW-AF, NIR-AF, PS-SLO, and PS-OCT. The results showed that intraretinal RPE migration was found in various stages of AMD, and occurred more commonly in eyes with drusenoid and serous PED.

There have been several studies reporting the frequency of HRF in various stages of AMD using standard OCT^7, 8, 10, 15^. In the present study, most of the HRF showed signs of RPE migration in the early to intermediate AMD, drusenoid PED, serous PED, and dry AMD groups. In contrast, the majority of HRF in the remission stage and the end-stage of exudative AMD groups lacked RPE migration. The extravascular blood constituent leakage that results from the disruption of the outer blood retinal barrier is an important pathological marker of active exudative AMD^43, 44^; these materials produce hyperreflectivities that appear as HRF. Lipoproteins, or lipoprotein-containing inflammatory cells, are thought to be possible sources of HRF under these conditions^14^. Thus, the specific AMD stage is an important parameter to consider when evaluating both the origin and pathogenesis of HRF. Similarly, the presence or absence of intraretinal RPE migration should be carefully evaluated in terms of the AMD stage.

In the present study, intraretinal RPE migration frequently occurred in the serous PED group. In some cases, a considerable amount of RPE cells migrated from the top of the PED, and this large migration appeared as “RPE eruptions” (Fig. 3c,d,e). Because RPE migration impairs the integrity of RPE layers, it is likely that such an extensive amount of migration is concomitant with the failure of RPE functioning. In eyes with serous PED, the amount of intraretinal RPE migration was significantly correlated with the PED volume. Mechanical stress induced by increased internal pressure in PED, as well as by the mechanical separation of RPE layers from the choriocapillary layer, could increase the PED size and is a possible cause of the intraretinal migration of RPE cells. In addition, inflammation, which is widely recognized as an important factor in the pathogenesis of exudative AMD, including serous PED^44^, can cause cellular stress that may weaken the adherence of the RPE to Bruch’s membrane, thus potentiating RPE cell motility^45–47^. Although the exact mechanism by which RPE migration occurs in serous PED is not yet fully understood, the presence of serous PED could be an important causative factor in intraretinal RPE migrations.

Another important finding was the detection of intraretinal RPE migration in cases of drusenoid PED. Drusenoid substances are extracellular accumulations of extracellular debris, lipids, and lipofuscin between the RPE and Bruch’s membrane^48–50^. Several studies have reported the presence of various inflammatory mediators in drusen^49, 50^. Inflammatory reactions caused by these mediators have been shown to induce the secretion of many chemokines and cytokines, which are chemotactic in nature^45, 46^. Notably, some studies have documented the positional association between drusenoid lesions and HRF^6, 10^, and reported that the presence of HRF can serve as a predictor of new atrophy^6–9^. Thus, intraretinal RPE migration could be used as an important predictor of the clinical course of drusenoid PED.

Our multimodal imaging approach allowed us to compare findings across different scanning methods. The projection of DOPU images obtained with PS-OCT showed similarities to the NIR-AF images. These similarities were also found using a depolarized light image obtained with PS-SLO. The presence of both depolarization and hyper NIR-AF of the melanin-containing cells has been previously confirmed in animal studies^51, 52^, and was thought to result from the distribution of melanin-containing cells. Although AF imaging can therefore provide important information about RPE pathology, the absence of topographic information remains an important limitation of this method. In contrast, PS-OCT can provide three-dimensional information about the source of depolarization. PS-OCT and NIR-AF images can complement one another because the three-dimensional capability of PS-OCT can provide topographical information about the NIR-AF images. The projection image of the DOPU anterior to the RPE (Figs 1g, 2g, 3g and 4g) excluded the influence of depolarization at the RPE and choroid, and was therefore useful in evaluating the distribution of HRF during RPE migration. Overall, the use of a multimodal imaging approach that included polarimetry imaging and AF imaging is a promising methodology for the evaluation of macular disease.

The detection of intraretinal RPE migration in the present study, which was based on the presence of melanin and lipofuscin, was limited by RPE pathological changes associated with AMD and by infiltrating inflammatory cells. During the epithelial-mesenchymal transition of RPE cells, which occurs in exudative AMD^53^, both melanosomes and lipofuscin decreased in density because of the multiplication of RPE cells due to the repeated dilution among daughter cells^54^. In addition, upon progression of cellular damage to the RPE in AMD, some RPE cells lose lipofuscin, which can cause the transition to subductive cells or melanocytic cells^2, 3^. In the present study, we may have missed such RPE cells undergoing the latter phase of epithelial-mesenchymal transition or the degeneration of RPE cells. Conversely, if inflammatory cells ingested simultaneously both melanosome and lipofuscin from disintegrating RPE cells, our imaging system might have misinterpreted these inflammatory cells as RPE cells, as stated above. However, inflammatory cells, including both melanin and lipofuscin, were less common in previous histopathological studies of AMD^2–5, 21, 22^. Additional histopathological studies will be required to resolve these limitations.

The present study has some limitations that should be discussed. Given the small number of patients in some groups, we could only evaluate some aspects of intraretinal RPE migration. Furthermore, we excluded active exudative AMD with apparent hard exudates due to the difficulty in identifying RPE migration in the area of depolarization in hard exudates^36^. Therefore, the present study only performed a limited evaluation of the active stage of exudative AMD. Additional procedural limitations were the time requirements to test a patient and the patient fixation stability. In the present study, 6.6 seconds were required for a single measurement using PS-OCT, despite using high speed 100 kHz OCT. This long measurement time caused motion artifacts and patient discomfort. Therefore, DOPU measurement results for small HRF should be interpreted with caution because of potential motion artifacts. To minimize the influence of motion artifacts on the findings, we based our conclusions on HRF depolarization results that were observed both by PS-OCT and PS-SLO. For future clinical applications, a reduction in measurement time will be important to resolve these problems. Another limitation was the lack of available topographic information when using SW-AF or NIR-AF imaging. Additionally, although HRF with low DOPU mass lesions in corresponding axial locations were excluded, there is still the possibility that we detected erroneous results due to the high-AF lesions around HRF. Furthermore, the HRF volumes were calculated based on the manual localization of HRF. Although the projection image of the minimum DOPU anterior to the RPE might be useful for automatic detection of HRF, the automatic segmentation of RPE lines in diseased retina was frequently problematic because of the erroneous identification of the RPE line and Bruch’s membrane^55^; thus, projection images anterior to the RPE were prepared using the manual segmentation of RPE lines. Further development of imaging techniques is required to resolve these important limitations. Finally, the present study evaluated HRF with RPE clusters that included RPE cells and possibly RPE fragments. Although these RPE clusters could be detected in repeated measurements (see Supplementary Figure S1), Augustin *et al*. reported that it is difficult to detect single melanin granules with PS-OCT^56^. Therefore, it is possible that some RPE cells were not detectable with PS-OCT.

In conclusion, the present study showed the clinical utility of multimodal imaging to evaluate RPE lesions in AMD patients. The topographical information of RPE cells was readily detected by PS-OCT, which provided *en face* information similar to that obtained with PS-SLO and NIR-AF. Multimodal imaging protocols that include polarimetry imaging and AF imaging are therefore effective tools for characterizing RPE changes during macular disease.

## Methods

### Subjects

We prospectively examined 155 eyes of 119 Japanese patients with AMD (88 males, 31 females; age range, 51–94 years; mean age, 74.8 years). The eyes were classified into six groups: early to intermediate AMD, drusenoid PED, serous PED, remission stage of exudative AMD, end stage of AMD with subretinal fibrosis, and dry AMD with geographic atrophy. Early to intermediate AMD was defined as AMD using categories 1, 2, or 3 of the Age-Related Eye Disease Study System^57^ without drusenoid PED. Drusenoid PED was defined as an RPE elevation by drusen that was at least 350 µm at the narrowest diameter. Serous PED was defined as serous PED without retinal or subretinal hemorrhage. The remission stage of exudative AMD was defined by the presence of a dry macula after an intravitreal anti-vascular endothelial growth factor injection for exudative AMD. The end-stage of AMD with subretinal fibrosis was defined as the presence of subretinal fibrosis tissue in the macula combined with a history of exudation. Dry AMD with geographic atrophy involved the center of the macula.

Eyes with severe cataracts or other eye diseases that could significantly compromise image quality were excluded from the study. The presence of hard exudates was an important obstacle for the detection of intraretinal RPE migration with PS-OCT imaging. In PS-OCT images, hard exudates were observed as a mass of congested HRF with depolarization^36^. In eyes with hard exudates, the discrimination of the RPE from the surrounding hard exudates was extremely difficult because of their intensity and polarization similarities. For this reason, the eyes with active exudative AMD with apparent hard exudates were excluded from the study.

The present study adhered to the tenets of the Declaration of Helsinki, was approved by the Institutional Review Boards of Tokyo Medical University, and was registered in a public database (UMIN000026307; http://www.umin.ac.jp/ctr/index-j.htm). The nature of the present study and the implications of participating in this research project were explained to all study participants, and written informed consent was obtained from each one before any study procedures or examinations were performed.

### PS-SLO

We used data from a commercially available polarimeter (GDx Nerve Fiber Analyzer; Laser Diagnostic Technologies, San Diego, CA, USA) for PS-SLO imaging. Linearly polarized light at 780 nm was used to scan the retina in a raster pattern, with a measurement area of 15° × 15°. The returning light was separated into two beams for an uncrossed polarized detector and a crossed detector. The acquisition time was 0.7 seconds for an image series. The depolarized light image was computed as the minimum value of light at each pixel returning to the crossed detector for all input polarization angles^26, 33, 34, 58, 59^. The intensities of the computed polarimetry images varied extensively, and as expected, the depolarized light images were too dark for subjective evaluation without further manipulation. For better visualisation, simple adjustment of the brightness of each image was applied with minimum to maximum intensities in the images, and then converted to a grayscale that ranged from 0–255 grayscale units (see Supplementary Figure S2).

### PS-OCT

For PS-OCT imaging, we used a multifunctional Jones-matrix OCT, built by the Computational Optic Group at the University of Tsukuba^27, 32, 60, 61^. This OCT system was based on swept-source technology, and operated at an axial scan speed of 100,000 A-scans/second, using a swept-source laser with a central wavelength of 1,048 nm. A raster scanning protocol with 512 A-lines × 256 B-scans covering a 6.0 × 6.0 mm region on the retina was used for volumetric scans. The depth range of each B-scan image was 2.1 mm. The acquisition speed of each volumetric measurement was 6.6 seconds/volume. The depth resolution was 6.6 μm in the tissue. For the PS-OCT measurements, B-scan measurements were repeated four times at a single location. DOPU was calculated to evaluate the depolarization or the polarization scramble of the tissue^35^. In our analyses, DOPU with Makita’s noise correction^28^ was utilized and was computed using a 3 pixel (transverse) by 3 pixel (depth) kernel. Composite DOPU B-scan OCT images, in which the area of low DOPU (<0.8) was overlaid on the standard OCT B-scan image with red color, were created to specify the location of the depolarization observed in the standard OCT image. From each volume data set, we calculated an *en face* projection image of the minimum DOPU along the whole depth. We also calculated an *en face* projection image of the standard OCT images. To evaluate the HRF distribution, the anterior boundary of the RPE border was manually delineated in each standard OCT B-scan image, and *en face* projection images of the minimum DOPU anterior to the RPE were calculated with these segmentation lines.

### Multimodal imaging

We compared PS-OCT and PS-SLO images with color fundus images, NIR-AF images, and SW-AF images. Color fundus images were captured using a fundus camera combined with a Topcon 3-D OCT-2000 (Topcon, Tokyo, Japan). Both NIR-AF images (787 nm excitation) and SW-AF images (488 nm excitation) were obtained using the HRA2 (Heidelberg Engineering, Heidelberg, Germany), and were saved in 8-bit grayscale images. For the comparison of multiple image types, retinal vascular architecture was manually aligned across images using image processing software (Adobe Photoshop CS5; Adobe Systems, San Jose, CA, USA). PS-OCT volumes without significant motion artifacts were used for this registration. The presence of HRF with RPE was defined by the presence of a low DOPU at HRF for PS-OCT images, and the simultaneous presence of depolarization in PS-SLO and hyper-AF for both NIR-AF and SW-AF images at corresponding locations. HRF with low DOPU mass lesions in corresponding axial locations were excluded. The presence of HRF with or without RPE was evaluated by two blinded observers (R.T. and T.I.). In the case of discrepancies, a third observer (H.G.) acted as a referee and helped reach consensus.

### The volume of intraretinal RPE migration and PED

**Figure 7:**
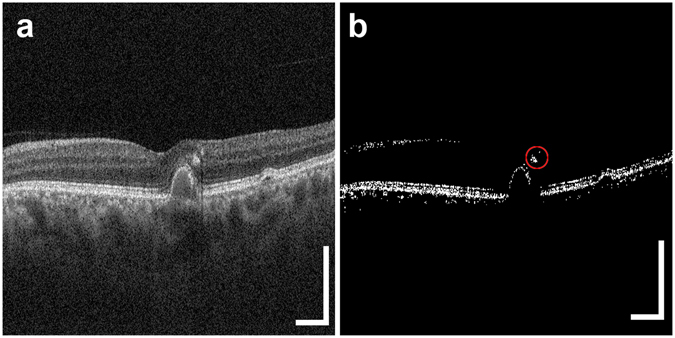
Image processing used to measure the areas of HRF with RPE. Original standard OCT B-scan image (**a**). Image after application of the Shanbhag filter (**b**). The circle with a red line designates the area with HRF. The scale bars represent 500 μm × 500 μm. All abbreviations used are as defined in the figure legend for Fig. 1.

To evaluate the HRF volumes with RPE, each standard OCT B-scan image was binarized using a Shanbhag filter, and the HRF loci with RPE were manually selected in each binary image (Fig. 7). The HRF areas with RPE were measured with image processing software (Fiji^62^), and the total HRF volume in each eye was calculated by summing the HRF areas with RPE in the B-scan image series. Hence, the total HRF volume per eye was calculated as 11.7 µm × 23.5 µm × 3.6 µm × number of pixels within the segmented HRFs. The mean HRF volume was calculated as the average of the total HRF volumes measured in all eyes for each group. For eyes in the drusenoid PED and serous PED groups, the PED volume was calculated with a series of B-scan images. The inner boundary of the PED was manually selected in each B-scan image and the inner area of the PED was measured with image processing software (Fiji^62^). The total PED volume was determined by summing the volumes of individual segments using the Cavalieri principle of stereological analysis^9, 63^.

## Electronic supplementary material

Supplementary Figures
