# Supplementary material for: Evaluation of intraretinal migration of retinal pigment epithelial cells in age-related macular degeneration using polarimetric imaging
Source: Sci Rep. 2017 Jun 9;7:3150. doi: 10.1038/s41598-017-03529-8 (PMC5466639; doi:10.1038/s41598-017-03529-8)
Supplement: Supplementary file 1 — Supplementary Figures [file 41598_2017_3529_MOESM1_ESM.pdf]

Evaluation of intraretinal migration of retinal pigment epithelial cells in age-related macular degeneration using polarimetric imaging

Masahiro Miura, Shuichi Makita, Satoshi Sugiyam, Young-Joo Hong, Yoshiaki Yasuno, Ann E. Elsner, Shigeo Tamiya, Rintaro Tsukahara, Takuya Iwasaki and Hiroshi Goto

Supplementary Figure S1
Example of repeated measurements.

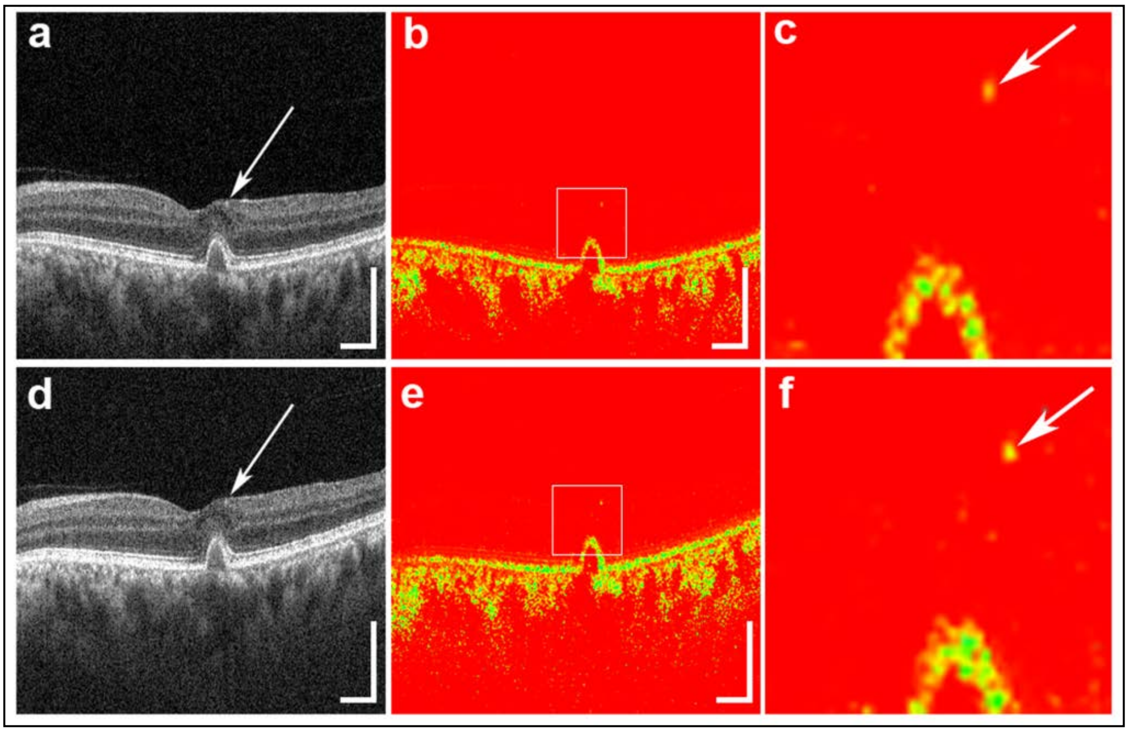

Example of repeated measurements. The first (a, b, c) and second (d, e, f) measurements were carried out in a sequential manner. Standard OCT images showed tiny HRF (white arrows) in both the first and second measurements (a, d). The DOPU B-scan images (b, e). White lines indicate the areas used in the high magnification images (c, f). High magnification DOPU B-scan images (c, f) clearly show depolarization at the tiny HRF (white arrows) for both measurements. The scale bars represent 500 μm × 500 μm.

Supplementary Figure S2
Brightness adjustment for PS-SLO depolarized light images.

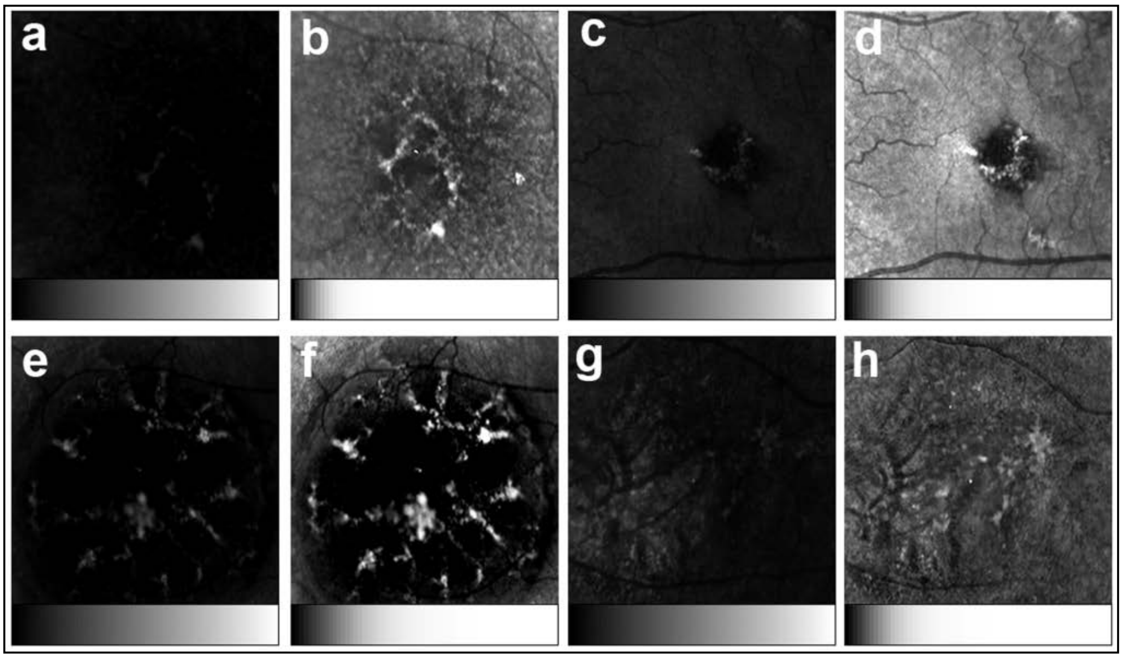

Brightness adjustment for PS-SLO depolarized light images seen in figure 1 (a, b), 2 (c, d), 3 (e, f), and 4 (g, h). Original images (a, c, e, g). Images after brightness adjustment (b, d, f, h).
